# A New Outcomes-Based Payment Plan From a Chinese Company to Improve Patient Affordability of CAR-T Product

**DOI:** 10.34172/ijhpm.8543

**Published:** 2024-08-20

**Authors:** Si-Li Zheng, Xiao-Qing Zhang

**Affiliations:** ^1^Department of Pharmacy, The International Peace Maternity and Child Health Hospital, School of Medicine, Shanghai Jiao Tong University, Shanghai, China.; ^2^Shanghai Key Laboratory of Embryo Original Diseases, Shanghai, China.

## Dear Editor,

 Chimeric antigen receptor (CAR)-T cell immunotherapy is rapidly transforming the landscape of cancer care, producing significantly good remission rates in patients with hematological cancers.^1^ Despite the remarkable outcomes in clinical trials, many patients cannot access this treatment due to high costs.^2^ This results in patients out of reach of desired medications (CAR-T drugs) and companies incurring revenue deficit, significantly curbing the positive social effects of the innovative drugs. Therefore, novel payment strategies are imperative to allow broader access to these new therapies.

 Recently, a pharmaceutical company in China, Fosun Kite Biotechnology, announced a new outcomes-based payment plan to improve patient affordability of its CAR-T product Axicabtagene Ciloleucel injection (FKC876) (Chinese trade name: 奕凯达, an autologous CD19-directed CAR-T cell therapy manufactured in China under a license to Yescarta^®^).^3^ A lot of attention in society has been attracted by this plan, according to which, eligible patients who do not achieve a complete disease remission within 3 months after CAR-T cell infusion will receive a refund of up to 600 000 RMB, which is approximately US$ 84 105.

 This novel business model, wherein payment is made “according to efficacy,” is industry-recognized as a “variant of massive discounting.”^4^ While some insiders opine that this move represents a kind of “self-rescue” effort by the CAR-T pharmaceutical company.^4^ For patients, facing an affordability problem of CAR-T drugs at present, would be more hopeful to reach these drugs in the future under this innovation of drug payment within China.

 From 2021 to now, five CAR-T cell products (Table) have been approved by China National Medical Products Administration.^5^ The current list pricing ranges between 0.999 million and 1.29 million RMB per infusion, far more expensive than standard-of-care therapies. In addition to the drug cost of the CAR-T cell products, other costs including procedure costs and facility costs significantly add to the final price tag. Given the staggering cost of CAR-T cell therapies, and the fact that a significant fraction of patients achieving an initial complete response with CAR-T will ultimately relapse,^6^ many patients are reluctant to adopt this treatment, worrying about loss of both life and money.

**Table:** Summary of CAR-T Products Approved in China

**CAR-T Products**	**Trade Name**	**Indications**	**Company**	**Approval Time**	**Price Per Infusion (RMB/US Dollars)**
Axicabtagene Ciloleucel Injection	奕凯达 (Yikaida®)	Adult r/r LBCL after two or more lines of systemic therapy, including DLBCL not otherwise specified, PMBCL, high-grade B-cell lymphoma and DLBCL arising from follicular lymphoma Adult r/r LBCL who failed first-line immunochemotherapy or relapsed within 12 months	Fosun Kite	2021-06-22	￥1.2 million/~US$ 168 211*
Relmacabtagene Autoleucel Injection	倍诺达 (Carteyva®)	Adult r/r LBCL after two or more lines of systemic therapy	JW Therapeutics	2021-09-01	￥1.29 million/~US$ 180 827*
Equecabtagene Autoleucel Injection	福可苏 (FUCASO®)	Adult RRMM after three or more prior lines of therapy and progressed, including a proteasome inhibitor and an immunomodulatory agent	IASO Biotherapeutics	2023-06-30	￥1.166 million/~US$ 163 445*
Inaticabtagene Autoleucel Injection	源瑞达	Adult r/r B-ALL	Juventas Cell Therapy	2023-11-07	￥0.999 million/~US$ 140 035*
Zevorcabtagene Autoleucel Injection	赛恺泽 (Zevor-cel®)	Adult RRMM after at least three lines of therapy and progressed, including a proteasome inhibitor and an immunomodulatory agent	CARsgen Therapeutics	2024-02-23	￥1.15 million/~US$ 161 202*

Abbreviations: r/r LBCL, relapsed or refractory large B-cell lymphoma; DLBCL, diffuse large B-cell lymphoma; PMBCL, primary mediastinal large B-cell lymphoma; RRMM, relapsed or refractory multiple myeloma; r/r B-ALL, relapsed and refractory B-cell acute lymphoblastic leukemia; CAR-T, Chimeric antigen receptor-T. * Using exchange rate: $1 = ￥7.1339.

 This outcomes-based payment plan launched by Fosun Kite will make patients more confident in CAR-T products. For them, CAR-T cell therapy is the most promising therapy at present offering potentially curative treatment for hematological tumors. Even if complete remission cannot be achieved, certain treatment benefits would be obtained with a refund of up to 600 000 RMB, which will alleviate the financial burden on families. By this way, the patients’ hesitation of CAR-T products is reduced, adding to the possibility of taking this therapy. For clinicians and researchers, the larger number of patients participated in this therapy will enable broader clinical data collection of CAR-T products, especially supplementing data about long-lasting clinical benefits, which is quite important to further improve the drug efficacy, and investigate adverse reactions as well as the mechanisms and coping strategies.

 It should be pointed out that similar outcomes-based payment schemes for CAR-T cell therapies have been initiated in some other countries. For example, in Germany, rebates will be provided for patients who die within a time period after treatment with CAR-T products (Kymriah^®^ or Yescarta^®^).^7,8^ In Italy, payments for CAR-T cell therapies are made in instalments, as long as the agreed outcome(s) has been achieved and sustained.^7,8^ In Spain, reimbursements through outcomes-based, staged payments are made, provided that the patient has achieved and sustained a complete response to the treatment (Kymriah^®^) or key outcomes linked to survival have been obtained (Yescarta^®^).^7,8^ In the United States, Novartis offers an outcomes-based payment scheme for Kymriah^®^ (but only in the acute lymphoblastic leukemia indication) based on individual patient response at 30 days.^8^ Though innovative payments models may vary from one country to another, we believe that these are good attempts to balance the financial risks between patients and pharmaceutical companies, providing references to the payments of high-cost therapies.

 Being the country with the largest number of registered CAR T-cell trials presently,^9^ China has been striving to expand patient access to CAR-T cell therapies. On one hand, the government is seeking avenues to enhance safety and efficiency of CAR-T drug administration, such as the advancement of training medical professionals to facilitate the standardized use of the drugs. On the other hand, multi-party efforts have been made from governments at all levels, pharmaceutical companies, hospitals, charitable funds, commercial insurance companies, etc. to reduce the financial burden on patients. Currently, over 75 commercial insurance products incorporated the CAR-T cell therapy products into their protection initiatives. For instance, as early as July 2021, Ping An Insurance Company’s “i drug insurance” (Enhanced Edition) program augmented its original drug list by offering free CAR-T cell therapy treatment. Moreover, CAR-T products have been incorporated into at least 100 “Huimin Bao” insurances, which are the city customized commercial medical insurances launched by local governments together with commercial insurance companies benefiting residents in a city. Taking Shanghai “Huimin Bao” (also called “Huhuibao”) as an example, the 2022 edition started to include CAR-T products into the insurance coverage, with an annual premium of 129 RMB and the highest medical insurance coverage up to 3.1 million RMB, in which the reimbursement ceiling for CAR-T products being 500 000 RMB. It was known that just within one month of the 2022 edition’s activation of “Huhuibao,” two claims for special drug treatment of CAR-T cells were processed, resulting in a total payout of 1 million RMB. Clearly, such policies have significantly augmented the accessibility of payment for CAR-T drugs, allowing both patients and pharmaceutical companies to reap benefits. However, the low premium in contrast to the high maximum reimbursement might raise concerns on the adverse selection problem^10,11^ in these “Huimin Bao” insurances. It should be noted that local governments have played a very crucial role by supporting, mentoring and actively participating in the development and operation of these insurances, making them a powerful tool to reduce residents’ financial burden of medical care. Firstly, the government provides necessary data support, which is important to the rational design and fair pricing of the insurance products. Secondly, high participation rates of the insurances can be reached *via* the government’s vigorous marketing publicity and promotion and allowing the use of personal medical insurance accounts to pay. Thirdly, strict supervision of insurance business is carried out, aiming to improve the service efficiency as well as the risk control ability of the insurance companies. Fourthly, efforts from multi-parties have been made to continuously optimize the security benefits of the insurances. At present, most “Huimin Bao” insurances are underwritten by over 2 commercial insurance companies, eg, 9 companies jointly underwrite the 2024 edition “Huhuibao.” These measures are of great significance in preventing the insurance products from falling into the dilemma of “death spiral.”^12^ Nevertheless, despite the above achievements, the sustainable and healthy development of “Huimin Bao” insurances still faces challenges and needs to be continuously improved and upgraded, which is widely concerned by the society.

 The initiative recently unveiled by Fosun Kite Biotechnology is considered as China’s first attempt to pay for CAR-T drugs based on efficacy. This measure is believed to bring greater confidence for patients to make good use of the medication in the future. Although it is currently unclear to what extent this innovation can broaden patient access to CAR-T drugs in China, the company’s behavior trying to provide more treatment opportunities for cancer patients is worthy of affirmation.

## Ethical issues

 Not applicable.

## Competing interests

 Authors declare that they have no competing interests.
